# Serum Autoantibodies: From Identification to Clinical Relevance

**DOI:** 10.1155/2013/382069

**Published:** 2013-03-20

**Authors:** Pietro Invernizzi, Xavier Bossuyt, Dimitrios P. Bogdanos

**Affiliations:** ^1^Liver Unit and Center for Autoimmune Liver Diseases, Humanitas Clinical and Research Center, Via Manzoni 56, 20089 Rozzano, Italy; ^2^Laboratory Medicine, University Hospitals Leuven and Experimental Laboratory Immunology, Department of Microbiology and Immunology, KU Leuven. Herestraat 49, 3000 Leuven, Belgium; ^3^Department of Medicine, Faculty of Medicine, School of Health Sciences, University of Thessaly, Biopolis, 41110 Larissa, Greece; ^4^Cellular Immunotherapy and Molecular Immunodiagnostics, Biomedical Section, Institute of Research and Technology Thessaly, 41222 Larissa, Greece; ^5^Institute of Liver Studies, King's College London School of Medicine King's College Hospital, Denmark Hill Campus, London SE5 9RS, UK

This special issue collates 22 papers (15 original and 7 reviews) reporting on various aspects of serum autoantibody research ranging from the identification of novel disease-related autoantigens up to the clinical significance of autoantibodies.

The recent assurance that indirect immunofluorescence (IIF) is the gold standard for anti-nuclear antibody (ANA) testing from the Task Force of American College of Rheumatology (ACR) [1] has led clinical immunologists to revisit the urgent need for automated IIF platforms, which could guarantee the robust and accurate interpretation of ANA patterns [2, 3]. In this special issue, three papers (two original and one review) have assessed the applicability of automated indirect IIF platforms for autoantibody pattern recognition. This topic is emerging as one of the most important advances in recent years that could pave the way for a new era on IIF testing in autoimmune diagnostics. 

D. Roggenbuck et al. have provided an up-to-date overview of the data so far provided using the AKLIDES system and critically discuss the *pros* and *cons *of digital automated indirect IIF platforms for autoantibody detection in systemic rheumatological conditions. J. Voigt et al. report original data on the evaluation of ANA on HEp-2 cells using the EUROPattern Suite automated processing and interpretation system. This is the first time that original data on the performance characteristics of this platform have been published in the form of a full-length paper. Concordant results between visual and automated evaluation by the EUROPattern reached 99.4%. This supports the notion that a precise and reproducible differentiation of positive and negative samples tested by HEp-2 cell lines is met by the developed systems. Discrepancies between manual and automated pattern recognition is largely limited to serum samples with mixed ANA patterns, but the developers of those platforms assure the reader that they will soon overcome the current limitations. J. Damoiseaux et al. evaluated the first automated anti-neutrophil cytoplasmic antibodies pattern recognition system developed using the AKLIDES platform. Discrimination of C-ANCA and P-ANCA is satisfactory but the sensitivity on ethanol-fixed neutrophils needs further improvement. 

A considerable proportion of apparently healthy individuals have IIF-detected ANA targeting the dense fine speckles 70 (DFS70) antigen. The clinical interpretation of positive anti-DFS70 antibody associated pattern (DFS) tests has emerged as one of the most important problems that routine laboratories are faced with, as it clearly influences the specificity and the positive likelihood of the ANA tests. In their review paper, M. Mahler and M. J. Fritzler discuss this topic and also describe a novel immunoabsorption method that can block anti-DFS70 antibody reactivity. An original paper by M. Miyara et al. analyzed the clinical value of anti-DFS70 antibodies in patients subjected to routine ANA testing by IIF and concluded that systemic autoimmune rheumatic disorders are less prevalent in patients with the DFS pattern compared to patients with homogenous or other ANA IIF patterns.

R. Yoshimi et al. review the current data surrounding the clinical relevance and the pathogenic significance of anti-Ro/SSA antibodies in systemic lupus erythematosus (SLE), Sjögren's syndrome (SS), and other autoimmune disease. An original paper by A. Wacker-Gußmann et al. provided data suggesting that foetal magnetocardiography can complement foetal echocardiography as a noninvasive approach to detect early electrophysiological signs of atrioventricular delay in foetuses exposed to maternal anti-SSA/Ro and anti-SSB/La antibodies. Anti-C1q antibodies have been detected in women with SLE who experienced failed pregnancy. An interesting study from Greek investigators demonstrated that anti-C1q antibodies cannot differentiate failed from normal pregnancies. The same investigators have found elevated levels of IL-15 compared to those with missed abortions and healthy intrauterine pregnancies, underlying the diagnostic potential of this marker. Another original study has assessed the clinical relevance of circulating glucocorticoid-induced TNFR-related protein ligand (GITRL) levels in patients with SLE. Chinese investigators found that GITRL levels positively correlate with anti-dsDNA titres and these levels were significantly higher in SLE patients with renal involvement and vasculitis compared to patients lacking these clinical manifestations. Another original article from a Chinese group has found that the titres of carbonic anhydrase III and IV autoantibodies are unusually high not only in patients with SLE and rheumatoid arthritis, but also in patients with other diseases including type 1 and type 2 diabetes. 

P. C. Teixeira et al. review the literature and discuss the current knowledge on the role of autoantibodies against apolipoprotein A-1 in cardiovascular diseases. T. Shirai et al. provide an overview on the existing knowledge on the biological significance of anti-endothelial cell antibodies for vascular lesions in autoimmune rheumatic diseases. They also discuss in great detail the principles and applications of identifying autoantigens expressed on cell surfaces, known as serological identification system for autoantigens using a retroviral vector and flow cytometry (SARF). A. Shimatsu and N. Hattori review the literature and discuss the diagnostic, clinical, and pathophysiological features of macroprolactinemia caused by high molecular mass complexes of prolactin with immunoglobulin G (IgG) and in particular anti-prolactin antibodies. In their research article, R. Fu et al. and co-investigators used a proteomic approach based on the membrane of bone marrow cells to identify the antigenic targets of autoantibodies detected in a subgroup of patients with immune-mediated pancytopenia.

A significant contribution comes from L. Mihályi et al. Those researchers provide a meticulous overview of their 40-year long clinical experience in the diagnosis and management of patients with autoimmune bullous dermatosis. In their research paper, A. Patsatsi et al. found that titers of anti-BP180 autoantibodies relate with disease activity in Greek patients with bullous pemphigoid, while a clinical study by Dalmády and colleagues provided data suggesting that autoantibodies targeting mutated citrullinated vimentin may assist the diagnosis of psoriatic arthritis. U. Lindberg et al. found that IgA ANCA specific for bactericidal/permeability-increasing protein (BPI-ANCA) identifies cystic fibrosis patients with adverse outcomesand discuss the pathogenic potential of these autoantibodies. 

Finally, four original papers report on the relevance of autoantibodies in autoimmune gastrointestinal and liver diseases. A. Antico et al. assessed the predictive value of combined testing of four serological markers in the diagnosis of autoimmune gastritis. These markers include anti-parietal-cell antibodies (PCA), anti-intrinsic factor antibodies (IFA), anti-*Helicobacter pylori* (Hp) antibodies, blood gastrin levels and are diagnostically useful in the classification of gastritis. Their predictive value is comparable to that of histologically assessed gastric biopsies. A British-German collaborative study found that the presence of Crohn's disease-specific pancreatic autoantibodies targeting the zymogen granule GP2 is largely limited to patients with ileal involvement. A. Kempinska-Podhorodecka et al. report on the influence of immunogenetics and their close interaction with humoral markers of liver autoimmunity. These researchers found that polymorphisms of genes involved in TNF-receptor signalling and in particular those of the TNF-receptor-associated factor 1 (*TRAF1*) do not confer susceptibility to primary biliary cirrhosis (PBC). However, the GG homozygotes have significantly higher titres of PBC-specific autoantibodies directed against gp210 autoantibodies compared to AA homozygotes, suggesting that this gene may immunoregulate the persistence of gp210-specific B-lymphocytes. Another intriguing original paper by C. Radzimski et al. reports the development of a recombinant cell-based IIF assay which allows efficient determination of autoimmune hepatitis-specific autoantibodies against soluble liver antigen. These autoantibodies are important for the confirmation of the diagnosis in patients with suspected autoimmune hepatitis and cannot be detected in routine laboratories by IIF.

The broad range of important topics discussed by the authors participating in this issue further underlines the notion shared by the editors of this special issue that serum autoantibodies remain as one of the most valuable tools for the study of autoimmunity [4–6].

*Pietro Invernizzi* *Xavier Bossuyt* *Dimitrios P. Bogdanos*
